# Understanding Factors That Support Community Health Worker Motivation, Job Satisfaction, and Performance in Three Ugandan Districts: Opportunities for Strengthening Uganda’s Community Health Worker Program

**DOI:** 10.34172/ijhpm.2022.6219

**Published:** 2022-04-18

**Authors:** Shivani Pandya, Mukesh Hamal, Timothy Abuya, Richard Kintu, Daniel Mwanga, Charlotte E. Warren, Smisha Agarwal

**Affiliations:** ^1^Department of International Health, the Johns Hopkins Bloomberg School of Public Health, Baltimore, MD, USA.; ^2^Population Council, Nairobi, Kenya.; ^3^Last Mile Health, Kampala, Uganda.; ^4^Population Council, Washington, DC, USA.

**Keywords:** Community Health Workers, Incentives, Uganda, Retention, Primary Healthcare, Community Health Programs

## Abstract

**Background:** Uganda’s community health worker (CHW) program experiences several challenges related to the appropriate motivation, job satisfaction, and performance of the CHW workforce. This study aims to identify barriers in the effective implementation of financial and non-financial incentives to support CHWs and to strengthen Uganda’s CHW program.

**Methods:** The study was implemented in Uganda’s Lira, Wakiso, and Mayuge districts in May 2019. Ten focus group discussions (FGDs) were held with 91 CHWs, 17 in-depth interviews (IDIs) were held with CHW supervisors, and 7 IDIs were held with policy-level stakeholders. Participants included stakeholders from both the Ugandan government and non-governmental organizations (NGOs). Utilizing a thematic approach, themes around motivation, job satisfaction, incentive preferences, and CHW relationships with the community, healthcare facilities, and government were analyzed.

**Results:** CHWs identified a range of factors that contributed to their motivation or demotivation. Non-monetary factors included recognition from the health system and community, access to transportation, methods for identification as a healthcare worker, provision of working tools, and training opportunities. Monetary factors included access to monthly stipends, transportation-related refunds, and timely payment systems to reduce refund delays to CHWs. Additionally, CHWs indicated wanting to be considered for recruitment into the now-halted rollout of a salaried CHW cadre, given the provision of payment.

**Conclusion:** It is imperative to consider how to best support the current CHW program prior to the introduction of new cadres, as it can serve to exacerbate tensions between cadres and further undermine provision of community health. Providing a harmonized, balanced, and uniform combination of both monetary incentives with non-monetary incentives is vital for effective CHW programs.

## Background

**Key Messages**

** Implications for policy makers**

- It is imperative to consider how to best support the current community health worker (CHW) program prior to the introduction of new cadres, as it can serve to exacerbate tensions between cadres and further undermine provision of community health services.
- Providing a harmonized, balanced, and uniform combination of both monetary incentives with non-monetary incentives is vital for effective CHW programs.
- The provision of incentives, both monetary and non-monetary, should have proper governance mechanisms to ensure that they are fair, sufficient, consistent, timely, and equitably distributed.
- Inconsistent and delayed delivery of promised incentives further results in demotivation and demoralization of CHWs.
- Fairness and equity are core components in ensuring CHW rights are not violated. All efforts must be made to ensure any costs incurred by them for their job are refunded.

** Implications for the public**

Community health workers (CHW) provide critical access to services for rural and hard-to-reach communities globally, including Uganda. However, they are often overworked, underpaid, and face challenges that undermine their motivation and performance. This directly affects the health of the most vulnerable communities. Advocacy to advance support for CHW programs can help advance the health of rural and remote communities. The study reaffirms the need to compensate CHWs appropriately for their efforts, as well as to invest in training, supervising, and supporting them. This study identifies incentives structures and barriers to implementing them that must be addressed in order to support and strengthen the CHW program in Uganda.

Community health workers (CHWs) have been the cornerstone of primary healthcare (PHC) for several low- and middle-income countries since the 1978 Alma Ata Declaration, which established their crucial role in PHC provision to achieve the Health for All goal.^1,2^ CHW generally refers to the category of frontline health workers who work in communities outside of fixed health facilities and have some type of limited formal training for the tasks they are expected to perform.^1^ CHW programs have been implemented globally to address ongoing health systems challenges of skilled health worker shortages and to increase health services coverage in remote areas.^1-3^ The critical role CHWs and PHC play in advancing universal health coverage has been emphasized in both the Sustainable Development Goals and the recent 2018 Astana Declaration.^4,5^ However, CHW programs continue to face high levels of attrition, challenges with insufficient and inadequate resources provided to CHWs, and lack of adequate supervisory support, which are to the detriment of CHWs and undermines their motivation, job satisfaction, and performance.^6,7^

 Uganda established its CHW program in 2001, adopting the Village Health Team (VHT) strategy that was recommended by the Uganda Health Sector Strategic Plan I to address the critical shortage of health workers.^6^ The VHT program is under the Ugandan Ministry of Health (MoH) and is funded in part by the government, as well as by a variety of donors who provide training and other support.^6,8^ A VHT is a group of 5-6 village health workers (VHWs); each VHW is selected by their village, and serves between 25-30 households.^9^ VHTs are provided an initial two-week basic training on health issues such as childhood illnesses; family planning; antenatal care; water, sanitation, and hygiene; and are mobilized in the communities as volunteers.^8^ They are responsible for providing a range of services at the community and household levels, which includes health promotion and education, mobilization of communities for utilization of health services, case management for specific infectious diseases, referrals to health facilities and follow-ups, and the distribution of health commodities. The 2010 VHT Strategy and Operational Guidelines report, published by the Ugandan MoH, identified an initial incentive package for VHTs, which comprised of trainings, certificates, a commissioning ceremony, badges, t-shirts, bags, job aids, and registers.^9^ The report also indicated that VHTs should be provided a minimum of 10 000 Ugandan shillings (USh) (~US $5 at time of the report) per month.^9^ However, in practice, such incentives have been inconsistently implemented, and are varying across districts based on the level of support that is available from various global and local non-governmental organization (NGO)-partners.^6^

 A comprehensive national assessment of VHTs in 2015 estimated that over 179 000 VHTs were trained since the program’s inception and were operating in all 112 districts.^6^ However, about 30% of them were estimated to have already abandoned their positions.^6^ Critical gaps in the VHT program, including inadequate government funding, limited opportunities for training, lack of supervision and other support have resulted in sub-optimal performance of the program.^6,10-12^ The assessment resulted in a plan to roll out a new cadre of CHWs called the community health extension workers (CHEWs) at an estimated cost of US $102 million over 5 years.^13^ CHEWs would be based at the parish-level, serve as the supervisors of VHTs, would receive six months of formal training provided by the Ugandan MoH, and would be salaried.^12,13^

 Given the anticipated CHEW rollout, this study planned to better understand existing issues related to VHT compensation, so that it could inform the compensation plan for the new CHEW cadre. However, there was significant concern around the introduction of CHEWs, anticipated tensions between the two cadres, and the potential risk of further demotivating the existing VHT cadre.^12,13^ The CHEW policy had gained approval in January 2019 but was ultimately recalled due to “human resource gaps.”^14^ However, reforms to Uganda’s CHW program and the VHT cadre structure are still anticipated. This study provides insights into how to inform the structure of incentives that would best support VHTs, as well as any new cadres of non-voluntary CHWs planned for the future. It also aims to identify barriers in the effective implementation of these incentives.

 The provision of incentives, both monetary and non-monetary, is crucial to improve CHWs’ motivation, job satisfaction, and performance, which, in turn, affects their retention.^6,15^ Incentives – positive or negative, intrinsic or extrinsic – influence CHW motivation^7,16^ or their degree of willingness to exert and maintain an effort towards an organizational goal, as well as their job satisfaction and performance.^16,17^ Monetary incentives may include fixed salaries for those formally employed; event-based, monthly, or quarterly allowances; financial support in the form of educational support to CHWs’ children; or support for income-generating activities.^7,16^ Non-monetary incentives may include provision of bicycles, t-shirts, torches, and rain-coats. Additionally, ensuring that factors that serve to motivate them, such as access to regular supplies, appropriate supervision and training, and opportunities for advancement, are addressed may also be an incentive. Ormel et al identify these as ‘job enablers’ since they consider them as basic essential resources which the enabling environment – the health system – should provide in order to better support CHW motivation, job satisfaction, and performance.^16^

 While there is some research on the programmatic inputs that contribute to strong CHW programs,^10,18–22^ there is, however, a lack of such evidence on which strategies or combination of incentives would best motivate CHWs for their retention and effective performance.^15^ We, therefore, conducted a study within this context to better elucidate monetary and non-monetary factors that serve to motivate CHWs in Uganda, current incentives experiences amongst both VHTs (government CHWs) and NGO CHWs, and barriers impacting implementation of said incentives. A better understanding of how CHWs in Uganda are experiencing incentives provision provides key insights into how to motivate CHWs, improve their job satisfaction and performance, and increase CHW retention. This will help in creating a stronger and healthier CHW program that best supports CHWs and the communities they care for.

## Methods

###  Study Setting and Context

 The study took place in Uganda’s Lira, Mayuge, and Wakiso District; these districts were chosen after discussions with Uganda’s MoH and other stakeholders to provide perspectives from different regions and work settings in Uganda (see Figure). Lira was an intended CHEW pilot-site, Mayuge has hard-to-reach areas (ie, islands) that CHWs have to access, and Wakiso is an urban and higher cost-of-living district. This study is a part of a larger study conducted in Uganda, Kenya, Bangladesh, and Haiti looking at CHW incentive preferences to support their job motivation, satisfaction, and performance.^23^ The study aimed to understand CHW incentive preferences through focus group discussions (FGDs) and in-depth interviews (IDIs) with CHWs, CHW supervisors, and policy-level stakeholders.

**Figure:**
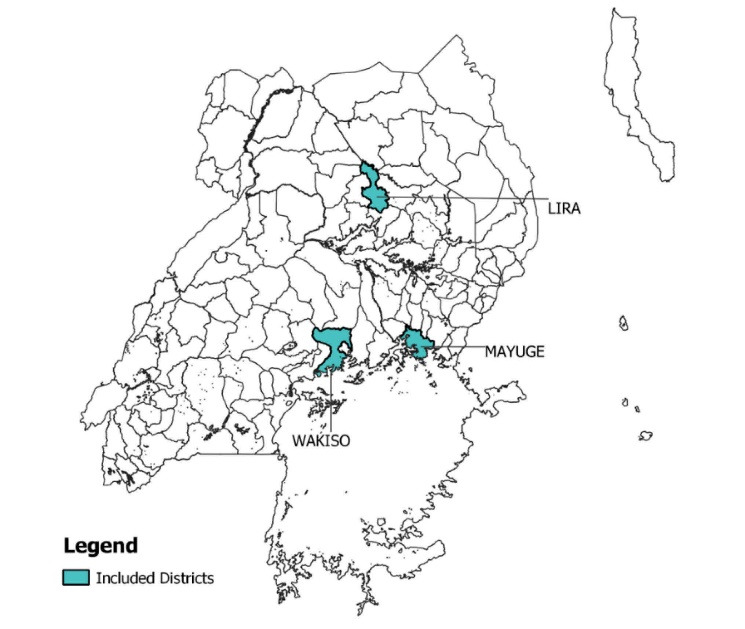
Included Uganda Districts (Lira, Mayuge, and Wakiso).

 CHWs included VHTs and NGO CHWs (ie, from BRAC and Living Goods) who are often referred to as community health promoters (CHPs). In some cases, VHTs (government-recruited and voluntary cadre) may also serve as CHPs (NGO-supported and typically receive some financial compensation). CHW supervisors included Health Assistants, Health-in-Charge/Facility-in-Charge, and District Health Educators. Policy-level stakeholders included representatives from the MoH and program managers for NGOs (ie, BRAC, Living Goods, Amref Health Africa).

###  Study Design and Data Collection

 FGDs were held with CHWs and IDIs were held with CHW supervisors and policy-level stakeholders. A semi-structured FGD/IDI guide was utilized to support the discussion; the same guide was used for both the FGDs and IDIs. Questions were asked around CHW incentive preferences, experiences around incentives that supported or inhibited CHW motivation and retention, relationships with the community and healthcare facilities, supervisory structures, and understanding of policy-level support (eg, around political goodwill and financial commitment towards the program) for CHWs. Ten FGDs were held with 91 CHWs, 17 IDIs were held with CHW supervisors, and seven IDIs were held with policy-level stakeholders in May 2019. See Table for a breakdown of participants by participant type and location.

**Table:** Total Number of Participants by District

**District**	**CHWs**	**CHW Supervisors**	**Policy Level Stakeholders**	**Total**
Lira	31	5	NA	36
Mayuge	30	7	NA	37
Wakiso	30	5	NA	35
National	NA	NA	7	7
Total	91	17	7	115

Abbreviations: CHW, Community health worker; NA, not available.

 Respondents were purposively selected; the study team worked with Uganda’s MoH to initially orient them to the study protocol and to facilitate access to community health units and PHC facilities. The study team also connected with district-level health leadership for their permission to contact the CHWs in 3-4 sub-counties within the district. The leadership helped facilitate contact with the CHW supervisors within the sub-counties who in turn informed the CHWs about the study. A convenience sample of CHWs were interviewed, based on who responded to the call for the study, and consented to participation. CHWs and CHW supervisors were recruited at the community health units and PHC facilities. Policy-level stakeholders were first identified by the study team and other relevant stakeholders, and then recruited for participation. All participants provided verbal or written consent before participating in the study. FGDs were held by two trained facilitators: one moderated the discussion and the second took notes; IDIs were held by one to two facilitators. All facilitators had at minimum a social science-related diploma, and were provided with training on the study design, protocol, and research ethics. They were fluent in the local dialects and have either grown up or had extensive lived experience in the district where they conducted the interviews. FGD and IDI duration ranged from one to two hours. Participants were all consented prior to their participation. The FGDs and IDIs were held in local language(s), audio-recorded, and subsequently transcribed and translated into English.

###  Analysis

 Thematic content analysis was utilized to analyze the data from the FGDs and IDIs, which comprised a mix of inductive and deductive coding.^24^ Analysis was conducted by a team of two researchers. The researchers were not involved directly with data collection; any areas of confusion or clarification was addressed by the Uganda-based team. The analysts familiarized themselves with the data and coding framework by reading and re-reading the transcripts. To assess interrater reliability, the analysts coded the data independently and then compared the codes. Any variations in coding were discussed to clarify definitions and arrive at a consensus. Emerging themes were given new codes. Coded data were compared and analyzed to provide a comprehensive picture of motivating factors and incentives and to summarize the similarities and differences in the perception and experiences of incentives amongst CHWs and between study sites. Data were coded and managed using QSR International’s NVivo 12 software.^25^ Results were disaggregated by CHW type (ie, VHTs and CHPs) and location to understand challenges and differences in experiences that were specific to specific cadres and/or locations.

## Results

 We discuss CHW preferences for monetary and non-monetary incentives, as well as barriers experienced in implementation of incentives by CHWs. Under each section, we discuss both facilitators contributing to CHW motivation, job satisfaction, and performance, as well as the challenges that have been experienced due to inadequate implementation of planned incentives.

###  Monetary Incentives

 While VHTs do not receive a salary from the government, CHPs – depending on the NGO that supports them – do receive monthly allowances. However, these were not always provided consistently, often due to upstream funding constraints.

 “*We were supposed to give them [CHWs] a monthly allowance of 80 000 USh. Some money to repair their bicycles and a lot of things to support them. But we also had a challenge of funding, and money never came at the time we expected*” (NGO Stakeholder IDI).

 Further, CHPs reported that the amount provided for both their monthly allowances as well as reimbursements for work-related travel is largely insufficient: “*When we come here [for training], they give us 10 000 [ USh ], but you may find that the amount I use is more than that. I use a boda boda [bicycle or motorcycle taxi] of 2000 [ USh ] to [location A], then I have to come here to [location B]. I find myself using all the money they have given me is spent here*” (CHP/CHW FGD).

 With the previously anticipated rollout of a paid cadre of CHEWs, tensions around a salary have been further exacerbated amongst VHTs; the lack of a salary has detrimentally impacted their ability to provide for their families. Instead of providing routine community-based health services, some CHWs may only participate in short-term campaigns that are paid. One District-level supervisor reported that VHTs had asked for payment for services provided in their District, though they noted these were rare cases.

 “*As [CHWs], we need a salary since we go out in the villages to educate people, and it requires one to look clean while standing in front of these people. If given money to enable us to pay school fees, we can stand confidently in front of people. Even when asked for information, you can readily avail it to them. Sometimes, we stand in front of people when our minds are elsewhere, thinking about our homes, and our inappropriate clothes. As doctors of the village, we are missing something because we don’t receive [a] salary. We stand and get hungry yet at the need of the day, there is nothing to go back home with*” (VHT/CHW FGD).

 “*They [CHWs] only appear when there is a program with money. Sometimes where there is no money, just to help us mobilize, they don’t appear*” (Policy-Level Stakeholder IDI).

 “*It is a motivational [issue] because if these people come and they expect something like financial motivation, when they don’t get it, they end up doing other things – especially the young ones, sometimes they go and marry, others leave the villages and go to other places in other districts, others go and look for employment elsewhere, they say they cannot keep doing this yet they can go get employment somewhere, and if they are women, they will go to other places and marry. But majorly it is a financial motivation problem*” (Policy-Level Stakeholder IDI).

 “*Sometimes you wonder how they [CHWs] survive, and yet they are supposed to do that work. So sometimes they end up forcing some clients to pay some money in things […] These are rare cases, but it’s a challenge. If someone doesn’t have a salary, what do you expect?*” (Policy-Level Stakeholder IDI).

 Government capacity to financially support VHTs is variable. One district-level supervisor shares that there is a difference between being able to provide material resources versus a monthly stipend or salary, noting that there is a potential space to provide consistent payments to VHTs, even if it is not on a monthly basis, which can help provide further motivation to CHWs.

 “*You think [the] government can’t buy bicycles? They can. Government can manage. The incentives like t-shirts, government can manage uniforms. It’s a cloth of [ USh ] 5000. But what I know, [the] government can’t manage paying them every month. But they can be paid quarterly. If you pay somebody after every three months in a year, you pay him only four times. Even if you give him [ USh ] 50 000 quarterly, it makes them active*” (VHT/CHW Supervisor IDI).

 The previously planned but now halted rollout of the salaried CHEW cadre, who would have been a supervisory cadre for the VHTs, was also discussed at length. VHTs indicated wanting to be considered for the CHEW cadre, but noted that the priority for CHEW recruitment seemed to focus on identifying new recruits, thereby neglecting VHTs.

 “*The government to consider VHTs for the CHEW strategy rather than recruiting new people. We were trained, we are experienced, and we therefore qualify to be CHEWs. We work through many hardships and therefore qualify for that promotion, which we know has a renumeration package*” (VHT/CHW FGD).

 “*My suggestion is to train the existing VHTs to become CHEWs instead of training new people. Most of them have knowledge and they are known*” (Policy-Level Stakeholder IDI).

 Furthermore, given that salaries would be provided to the CHEW cadre, it raised the question of also providing VHTs with some form of compensation.

 “*And these people [VHTs] have always really tried to complain, ‘Really, why can’t they give us something, maybe people wanted to give the CHEWs 180 000 [ USh ], why can’t you give us maybe a quarter of that money you wanted to give those people?’ It has been their [VHTs] cry*” (Policy-Level Stakeholder IDI).

###  Non-Monetary Incentives

####  Recognition

 Recognition refers to the ways in which CHWs can be valued and supported in their work by the health system and the community. Recognition is a significant motivating factor for CHWs. CHWs are often referred to as *musawo*, or doctors, indicating the level of respect and credibility the community places in them. CHWs also receive recognition through their relationships with the healthcare facilities. While largely appreciated, CHWs indicated wanting tangible forms of recognition for their work, namely through certificates, which would denote the training and skills they have received, and through priority healthcare for themselves and their families. CHWs also discussed the importance of including them in meetings and other relevant events, as it would help further cement their value and importance in and for the community.

 “*We were well-trained, but we have nothing to present that fact. We have never received any document that suggests we completed any course. Let them [the government] give us certificates that show we were trained to carry out the roles of a VHT*” (VHT/CHW FGD).

 “*Us VHTs should not queue and wait for long at the health facility when we are sick or when we bring members of households seeking treatment*” (VHT/CHW FGD).

 In some cases, patients referred by the CHWs to the health facility were not treated well or returned to the community without receiving any treatment. Some CHWs described that healthcare facility staff tore up their referrals. Several CHWs also noted a few instances of healthcare facility staff mistreating patients. This undermines their ability to work effectively, discredits their value and contributions, and harms their relationships with the community:

 “*We refer these clients to health centers. But, if you refer, sometimes, we take our time to escort these patients right up to the health center. But they [staff] do ignore you. They don’t take it as serious that these are our helping hands*” (VHT/CHW FGD).

####  Transportation

 CHWs noted that the provision of transportation would help them to work more efficiently, as it would ease their ability to travel to communities/meetings and in mobilizing the community. Specifically, CHWs may receive allowances of around USh 5000 (US $2) for transportation reimbursements. These are neither consistently provided nor sufficient for their transportation needs: “*Sometimes, if transportation facilitation exists as motivation, it is in small quantities*” (CHW Supervisor IDI). This becomes further exacerbated when training sessions and/or meetings occur on short notice and payments are delayed, subsequently placing CHWs in vulnerable situations:

 “*Even the little money they give us, say like when we come for meetings, also takes too long to come. They call you in the evening or at night that there is a meeting the following day. You don’t have transport and sometimes the money that was meant for food at home is the one you end up using as a transport fee. But on reaching there, they only tell you that the transport refund will be sent on the phone, and it takes a whole month without being sent*” (VHT/CHW FGD).

 Some CHWs reported receiving transportation reimbursements and/or bicycles or motorcycles – largely from partnering NGOs.

 “*It will be better if we are given bicycles because there is a lot of work, we do in the villages. While you are still doing this, they say go do this, or that, so it becomes hard to walk on foot and be effective. Even people cannot notice you are working*” (VHT/CHW FGD).

 However, it is important to note that the provision of transportation should be coupled with support in covering the maintenance and repair of these vehicles, as the lack of provides further financial burden on CHWs who would utilize these vehicles for their work-related responsibilities: “*The VHTs asked us to help them in repairing them [motorcycles] but I told them that this financial year, we did not have the budget for them*” (VHT/CHW Supervisor IDI).

####  Identification

 Identification refers to the ways in which CHWs are identified in their role as healthcare workers by the community and healthcare facilities. CHWs primarily received branded t-shirts, from both the government and from partnering organizations. Identification was noted to help facilitate their recognition as CHWs within communities and healthcare facilities.

 “*Because the clinical officers may not master [recognize] them all, then when they [CHWs] are in t-shirts, they [clinical officers] give you a referral, come in, and they give you service faster. So those and others motivate them*” (VHT/CHW Supervisor IDI).

 Some CHWs had been provided with the Global Alliance for Vaccines and Immunisation (GAVI)-branded bicycles in the past, which was reported to be motivating for them:

 “*When I was still working in [location], there were bicycles provided through GAVI. So, we used to give one person [a bicycle] for every five [CHWs]. This used to motivate them to ride a bicycle that has labels of [CHW] for a certain village. This motivates them so much*” (VHT/CHW Supervisor IDI).

####  Trainings

 CHWs reported receiving trainings from the government or partner NGOs charged with implementing community-based programs. However, in-service training is typically inconsistently provided, often occurring to support specific campaigns, rather than to facilitate comprehensive training of CHWs in the areas where they routinely provide services.

 “*Government and organizations should help train us. Some of us have served the community for so long but still lack the expertise. […] If we are trained, we shall be able to do blood tests, diagnose, and give out drugs. We were so happy about that training […] but it has been in vain. [The] government has failed to implement it, would have even motivated us more that government is thinking about us*” (VHT/CHW FGD).

 CHWs discussed the need for trainings on a range of topics, which includes ante- and postnatal care, diarrhea, nutrition, HIV/AIDs, immunizations, family planning, among others. Several CHPs (from NGOs) reported that they received monthly refresher trainings, however the majority of CHPs did express the need for additional trainings as well. These trainings can help enable CHWs better educate and support their communities:

 “*We require trainings. The doctors should come out to our villages and teach us. In case of an emerging disease, they should educate us, and even the village people will understand if what we are doing is the correct thing*” (VHT/CHW FGD).

####  Provision of Working Tools

 CHWs indicated the need for protective gear (eg, gumboots, raincoats, umbrellas), carrying cases for their supplies (eg, medicines), job aids, and mobile devices to support their work responsibilities. In the past, CHWs noted that these tools were provided as a part of training sessions and would have branding from the partner organizations or the specific public health campaign. However, given that trainings occur infrequently, the CHWs do not receive these tools regularly.

 “*So other things that they have been giving them are umbrellas, gumboots, raincoats – but this is once in a while. There is no partner that says maybe every year I will give a raincoat. Actually, some give at the beginning when they [CHWs] want to come – maybe to entice them to enter the program, but then after they disappear. So, someone is like, ‘Eh they gave us a raincoat, but the raincoat became so old’*” (VHT/CHW Supervisor IDI).

 CHWs discussed the need for mobile devices, as they can contain job aids, collect data, and serve as a way to communicate with the community, healthcare facilities, and their supervisors:

 “*Mine was mentioning smartphone because transport like bicycle and smartphone can make our work easy or collect data easily, you know the world is advancing now, you cannot do anything with paper, you send everything through a phone*” (VHT/CHW FGD).

 Within this discussion, CHWs also noted the importance of also having solar panels to help charge these devices, given that there is not always electricity to provide that service in their homes and in the communities that they serve: “*But even these phones of ours, so hard to charge them and go to the field, it would be better, if possible, they get us solar panels […]. So, it becomes easier for us to charge our phones*” (CHP/CHW FGD).

## Discussion

 This study highlights several similar gaps that were reported by the comprehensive national assessment of VHTs in 2015, which pointed to sub-optimal performance of the cadre owing to poor investment and limited opportunities for training and support.^6^ Instead of strengthening the structure of the existing VHT cadre, this report contributed to the development of a new salaried cadre who would oversee VHTs – the volunteer cadre – further exacerbating tensions among this them. This research highlights changes in incentive structures that would help strengthen existing cadres, as well as knowledge that could be leveraged towards planning the incentive structures for any future CHW cadres in Uganda. Additionally, we identify barriers to effective implementation of these incentive structures, as well as variations in the types of incentives provided by governmental and non-governmental agencies.

 Commonly desired monetary incentives included salaries and periodic allowances such as transportation reimbursements; non-monetary incentives included recognition, transportation, identification, and the provision of working tools and trainings. This study identified several challenges with the timely and consistent receipt of financial compensation, as previously identified by other studies in Uganda.^10,11,26,27^ Other studies have reported that CHWs in several districts in Uganda, despite promises, did not receive quarterly allowances,^27^ or were often not compensated for expenditures associated with their work, resulting in them using their personal resources for their job-related responsibilities.^26^ The 2010 VHT Strategy and Operational Guidelines published by the Ugandan MoH aimed to address these challenges through the provision of a national minimum incentives package for VHTs; this would provide a monetary allowance for lunch and travel whilst carrying out outreach and visits to health centers, and a minimum monthly stipend of US $5 (USh 10 000).^9^ However, this study suggests that these objectives have not been met, a decade later.

 The non-monetary incentives provided to CHWs in the study districts are misaligned with their needs to perform optimally in their role.^28^ In addition to the incentives, the study identified several factors that should be considered in the development of incentive structures. For example, if transportation such as bicycles are being provided, there is a need to account for maintenance and repair costs for transportation. Similarly, trainings should be accompanied by the consistent provision of working tools as well as commodities and supplies that support the CHWs in the activities. When CHWs are unable to provide the community with commodities (eg, contraceptives) that the community has come to expect, it undermines trust and further demoralizes CHWs. Our findings were similar to those conducted in Uganda as well as globally. CHWs cited that the lack of adequate transportation impacted ability to reach clients.^10,26,27,29^ Commodities stock-outs and inadequate supplies of medicine have also been major challenges reported by other Ugandan studies.^10,26,27,29^ Supervision or the lack thereof has been indicated to be a significant demotivator; while not significantly discussed in our study, other studies (including Ugandan studies) have suggested the importance of supportive supervision to help support CHW motivation and performance.^27,29,30^ CHWs in our study often highlighted issues with recognition and disrespect from the health facility staff, which affected their credibility with the community, as also identified by other studies in Uganda.^26,27,29^ Further, CHWs in our study, as well as other studies both in Uganda^26,29^ and globally,^20^ described being included in meetings, trainings, and other relevant community functions and decision-making as being recognized by the health system and communities. Other forms of recognition include social rewards, appreciation from community members, preferential treatments for self or family and clients referred, being consulted on a range of problems, being asked to provide health services at the health center, and being selected for paid activities.^6,7,26^

 Large variations exist in the total salaries, transport reimbursements, and other compensations provided by the government and NGOs to CHWs. The study identified some differences in incentives experiences between VHTs and CHPs; as NGO-supported CHWS, CHPs often received higher salaries and additional trainings. We extrapolate that these variations at the community-level serve to further demotivate VHTs who are often less well compensated and have fewer opportunities for training and financial compensation from the government. Such donor-funded programs could potentially undermine the government community-based PHC programs in favor of short-term donor supported campaigns that are typically better funded and have higher payments for the health workers.^31^

 The World Health Organization (WHO) guideline on health policy and system support to optimize CHW programmes recommends that CHWs receive a financial package that is commensurate with their demands of their job.^15^ This study reinforces this recommendation, and further brings into question the challenge with requiring volunteers, who are mostly very poor, and often women, to devote not only their time and effort, but also, inadvertently, their personal financial resources to support their communities. The Ugandan government is committed to PHC – however, this is not reflected in the prioritization of health as a part of government expenditure. Even though official government policies intended to formalize a compensation package for VHTs, the implementation of this has not been proactively guided by the government. Rather, it has been dictated by the availability of donor support, which is often linked to vertical disease-specific programs. In our study, we observed that VHTs expectations for a compensation package was guided by their experience working with various short-term donor-funded programs. Repeatedly, they highlighted that these programs may sometimes pay well but often make promises that are not followed through, and incentive structures put into place ultimately end with the end of the programs.

 In response to the coronavirus disease 2019 (COVID-19) pandemic, the Government of Uganda launched a new strategy for community engagement for COVID-19 response, wherein it was stated that at least one VHT member per village will receive around USh 100 000 (~US $30) monthly.^32-34^ It is not yet clear what the impact of such strategy has been for both VHTs and the community, or if it will be sustained beyond the pandemic. The COVID-19 pandemic has further emphasized the critical role CHWs play in supporting the PHC and advancing health equity. However, it has also amplified the vulnerable positions CHWs are in, especially under overstretched healthcare systems. CHWs are placed at greater risk of contracting COVID-19 in order to connect and provide the community with necessary services; however, as seen pre-pandemic, they are not always being provided with adequate personal protective equipment and resources to work safely.^35^

###  Policy Implications

 In 2019, the Ugandan government intended to introduce a salaried CHW cadre (CHEWs), following the example of other countries which have a two-cadre community health model,^36^ with a volunteer and a paid cadre. However, in the context of Uganda, where VHTs have constituted the backbone of the community health programs for over two decades, introducing a new paid cadre, without addressing issues with the existing VHT cadre, could backfire. The 2015 national VHT assessment reported an overwhelming demand for a harmonized and regular financial form of incentive for VHTs, as well as provision of training and supervision.^6^ Even if a new cadre is deployed at a supervisory level, the role of VHTs is crucial in supporting communities. Without concerted efforts to address the lack of harmonized support for VHTs, there is a high possibility that the relationship between the VHTs and a new paid cadre would not be cordial, further undermining community health.^12^

 The Ugandan government has stipulated various forms of monetary and non-monetary incentives for VHTs; however, it lacks clarity on what combination could best support VHTs, how it should be administered, and by whom.^6^ A complementary study conducted by this research team in Uganda recommended that VHTs are willing to accept a decrease in salary of USh 31 240 (US $8.5) in exchange for identify badges.^12^ This study highlights several non-financial incentives and barriers to implementing them that should be addressed to better support and motivate VHTs, and ameliorate their job satisfaction and performance.^12^ Given the relatively low government budget for PHC, it is important to carefully consider this balance of financial compensation along with non-financial incentives, which can potentially serve to motivate the community health workforce, and be financially sustainable for the Ugandan government. Insights from this and other studies also highlight several factors that must be considered (eg, maintenance of bicycles) for effective implementation of incentives provided, and checks and balances that should be in place to ensure equitable distribution.

 Lastly, there is a lack of national- and local-ownership of and commitment to the VHT program, which has led to it being underfunded and poorly managed.^10^ Nearly 45% of Uganda’s healthcare expenditure is funded through external, non-governmental sources.^37^ The VHT program is also largely dependent on NGOs, who are ultimately obliged to donor priorities and often have temporary, unpredictable commitment to the VHT program.^10^ Our study shows how several development partners and donors have parallel payment systems for VHTs. These need to be harmonized as much as possible so that they can be rolled out as a unified government strategy, instead of the current fragmented payment structures. Long-term funding options should considered at every level of the health system to encourage stronger and wider health systems strengthening.^13^ Uganda’s MoH sought to address this through the CHEW strategy, by bringing together local and global stakeholders to facilitate multi-stakeholder dialogue and commitment.^12-14^ Plans to strengthen the community health workforce in Uganda must include the importance of a harmonized and uniform incentive package for all CHWs, such that it is equitably structured across cadres of healthcare workers and standardized across all CHWs, as well as accountability mechanisms to ensure it is systematically and consistently implemented in practice.

###  Strengths and Limitations

 The effectiveness of different types of CHW incentives may depend on contexts, eg, the public versus the non-governmental sector CHWs, salaried versus non-salaried, or other locally-specific contexts. Although this study did disaggregate data by CHW type and location during analysis, we were not able to make any context-specific distinctions of the incentive preferences. One potential reason we did not note much difference between CHWs could be due to the fact that our study team prioritized VHT FGDs, conducting six FGDs amongst 60 VHT participants, and four FGDs amongst 31 CHP participants. Further, we did not collect demographic data around gender and age from our participants, which limits the opportunities to understand how gender and age play a role in CHW experiences.

 This study has been limited to three districts of Uganda, which might constrain the generalizability of the study findings to the broader national context. The selected districts, however, are from different regions that ensure a greater regional representation. Moreover, our study findings have been consistent with other studies in Uganda from several other districts and regions that reaffirm our study findings but also enhance the generalizability to a larger extent. Although the study was intended to assess incentives and explore tensions with the previously planned rollout of the CHEW cadre; at the time of implementation, the CHEW plan was held back.^14^ Despite this, the results of our study remain important in considering avenues of support for the existing cadres of CHWs.

## Conclusion

 In this study, we explored the perspectives of CHWs and other policy stakeholders to identify considerations for structuring incentive packages to strengthen the CHW program in Uganda. We reaffirm the need to develop a harmonized national-level plan to strengthen the VHT cadre through adequate compensation and health systems support, as well as to conduct multi-stakeholder consensus-building to harmonize funds so that a unified government strategy for CHWs can be implemented, instead of the current fragmented approach.

## Acknowledgements

 The authors thank the Government of Uganda’s MoH for the support in the design and implementation of the study. The authors would also like to thank the CHWs, their supervisors, and the policy-level stakeholders for providing their time and sharing their experiences towards contextualizing and identifying ways to best support CHWs and CHW programs in Uganda. We thank the data collection team for the excellent insights on contextualizing the survey to the different districts where the study was conducted.

## Ethical issues

 The research protocol was approved by the Population Council’s Institutional Review Board (PC IRB 872) and Makerere University College of Health Sciences’ Institutional Review Board (Protocol 657).

## Competing interests

 Authors declare that they have no competing interests.

## Authors’ contributions

 SA and RK conceptualized the study. SA, RK, and TA developed the study protocol. SA, RK, TA and DM led the field data collection. SP and MH conducted the analyses for this study. CEW is the overall PI for this multi-country study. SP, MH, and SA wrote and finalized the paper, with TA, RK, DM, and CEW providing revisions and feedback. All named authors had a role in the design, implementation, or analysis of this study.

## Funding

 This study was supported by a grant from the Bill & Melinda Gates Foundation. The funders had no role in the design and implementation of the study.
